# *In Vitro* Activity of Plant Extracts and Alkaloids against Clinical Isolates of Extended-Spectrum β-Lactamase (ESBL)-Producing Strains

**DOI:** 10.3390/molecules16075453

**Published:** 2011-06-28

**Authors:** Guo-Ying Zuo, Fan-Yan Meng, Jun Han, Xiao-Yan Hao, Gen-Chun Wang, Yun-Ling Zhang, Qing Zhang

**Affiliations:** 1 Research Center for Natural Medicines, Kunming General Hospital, PLA, Kunming 650032, China; Email: zhangyunling@126.com (Y.-L.Z.); kmz@263.net (Q.Z.); 2 School of Pharmacy, Guiyang Medical College, Guiyang 550004, China; Email: mfy_0@163.com (F.-Y.M.); 3 School of Basic Medicine, Yunnan Traditional Chinese Medical College, Kunming 650500, China; Email: hanzjn@126.com (J.H.)

**Keywords:** antibacterial plant extracts, ESBLs, 8-hydroxydihydrosanguinarine, 8-hydroxydihydrochelerythrine

## Abstract

The antibacterial activity of 80% ethanol extracts of 10 medicinal plants collected in Yunnan (Southwest China), was tested against clinical isolates of extended-spectrum β-lactamase (ESBL)-producing strains. Their MIC values ranged between 1.56-12.50 mg/mL. The most active plant extract was *Chelidonium majus* L. (MIC = 1.56 mg/mL). Two potent isoquinoline alkaloids, 8-hydroxydihydrosanguinarine and 8-hydroxydihydrochelerythrine, were identified as the major active principles through bioassay-guided fractionation and identification of the active ethyl acetate fraction from *C. majus*, with minimum MIC/MBC values of 15.63/62.50 μg/mL.

## 1. Introduction

Extended-spectrum β-lactamases (ESBLs) are enzymes produced in some Gram-negative bacilli that mediate resistance to extended-spectrum antibiotics like cephalosporin and aztreonam. The first clinical isolates expressing acquired ESBLs were identified in Germany in 1983 [1]. ESBLs have the ability to hydrolyse and cause resistance to various types of the newer β-lactam antibiotics, including the expanded-spectrum (or third-generation) cephalosporins (e.g., cefotaxime, ceftriaxone, ceftazidime) and monobactams (e.g., aztreonam), but not the cephamycins (e.g., cefoxitin and cefotetan) and carbapenems (e.g., imipenem, meropenem, and ertapenem). They are most common in clinical isolates of *Escherichia coli* and *Klebsiella pneumoniae* of the Enterobacteriaceae. The ESBL-producing multidrug-resistant (MDR) pathogens are the result of the selective pressure of currently used antibiotics and have been well recognized as a global nosocomial problem in recent years [2]. As plants can produce antimicrobial compounds to protect themselves from biotic attack, the search for antibacterial agents with different chemical structure and biological mode of action from plant sources is an alternative option in the fight against these microbes [3,4,5].

In the course of screening for active plant products against MDR bacteria, we paid special attention to the effects of Chinese medicinal plants on clinical isolates of ESBL-producing microorganisms. Here, we report the activities of ten plant extracts and two potent isoquinoline alkaloids 8-hydroxydihydrosanguinarine (hhS, **1**) and 8-hydroxydihydrochelerythrine (hhC, **2**), obtained through bioassay-guided fractionation and identification from one of the active extracts of *Chelidonium majus* L. (bai-qu-cai in Chinese, [6]).

## 2. Results and Discussion

As shown in Table 1, various potencies against MDR and the ATCC control of *E. coli* were demonstrated by the tested extracts, with inhibitory zone diameters (IZDs) of 11.0–21.0 mm and MIC values of 1.56–12.50 mg/mL, respectively. Most of the extracts were active not only against MDR but also non-MDR strains of *E. coli*, which suggested the different mechanisms of effect and their potentials as alternative sources for combating the increasingly troublesome problem of bacterial infections in hospitals [2].

**Figure 1:**
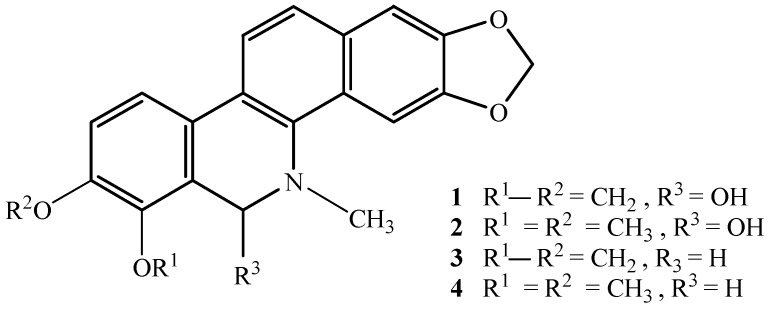
Structures of the four alkaloids (**1**: hhS; **2**: hhC; **3**: hS; **4**: hC).

Bioassay-guided fractionation and isolation of an active extract from *C. majus* led to the identification of the 8-hydroxylated benzo[c]phenanthridine alkaloids hhS and hhC as the major active compounds. They showed potent activity against both clinical MDR isolates and ATCC control strains of *E. coli*, with the low MIC of 15.63 μg/mL (Figure 1 and Table 2). Other compounds in the active extracts are under investigation.

From the results in Table 2, it is noted that though they were both phenotypic confirmatory ESBL-producing strains, the activity of hhS and hhC against such strains from *E. coli* were general much more active than those from *K. pneumoniae*. This could be attributed to the different resistance profiles of the two bacterial species with different genotypes [1,2].

**Table 1:** IZD (mm) and MIC (mg/mL) of the crude extracts and active fractions against selected strains.

No.	Species	Family	Speciesmen number	Part ^a^	*E*c ^b^		*E*c-MDR ^c^	
					IZD	MIC	IZD	MIC
1	*Chelidonium majus* L.	Papaveraceae	KUN201236	Wp	20.0	1.56	15.0	3.12
2	*Galla Chinensis* (Rhus chinensis)	Anacardiaceae	KUN61317	W	16.0	3.12	13.0	6.25
3	*Macleaya cordata* (Willd.) R. Br.	Papaveraceae	KUN201093	Ap	21.0	3.12	14.0	3.12
4	*Medinilla luchuenensis* C. Y. Wu et C. Chen	Melastomataceae	KUN372	R	13.0	3.12	11.0	6.25
5	*Michelia chapaensis* Dandy	Magnoliaceae	KUN12152	Rh	15.0	3.12	12.0	6.25
6	*Myrsine africana* L.	Myrsinaceae	HITBC032831	Ap	12.0	6.25	11.0	12.50
7	*Paeonia lactiflora* Pall.	Ranunculaceae	KUN 365353	R	16.0	3.12	13.0	3.12
8	*Sagentodoxa cuneata* (Oliv.) Rehd. et Wils.	Sagentodoxaceae	KUN3514	R	14.0	3.12	11.0	6.25
9	*Eurya yunnanensis* P.S. Hsu	Theaceae	YCP85965	Ap	11.0	12.50	NA ^d^	NA^ d^
10	*Viburnum foetidum* Will. Var. foetidum	Carprifoliaceae	KUN287	Ap	NA ^d^	NA^ d^	NA ^d^	NA ^d^
11	*C. majus*	Papaveraceae	KUN201236	Fr-E	19.0	1.56	17.0	3.12
12	*C. majus*	Papaveraceae	KUN201236	Fr-E1	26.0	0.39	20.0	1.56
13	*C. majus.*	Papaveraceae	KUN201236	Fr-E2	22.0	0.78	19.0	1.56

^a^ Ap: aerial parts; F: fruits; R: roots; Rh: rhizome; Wp: whole plants; W: wart, Fr-E: ethyl acetate fraction; Fr-E1 and Fr-E2,: two sub-fractions of Fr-E; ^b^ Ec: *E. coli* (ATCC25922); ^c^ *E*c-MDR: A total of five selected isolates of ESBLs-producing strains were tested; ^d^ NA: not active at concentrations up to 50 mg/mL.

*C. majus* has a long history in Chinese and European herbal medicine [6,7] as being useful for the treatment of many diseases. The present study reveals its *in vitro* anti-MDR activities against clinical isolates of ESBL-producing strains for the first time as far as we are aware [7,8]. It was found that among the four tested alkaloids the 8-hydroxylated ones were generally more active than the non-hydroxylated alkaloids. This type of alkaloids has been previously demonstrated to possess activity against clinical isolates of methicillin-resistant *Staphylococcus aureus* (MRSA) and resistant clinical yeast isolates [9,10]. Hence, they show broad-spectrum antimicrobial properties. However, to assess the efficacy and safety of these extracts and pure compounds, further *in vitro* and *in vivo* pharmacological assays are needed.

**Table 2:** MICs and MBCs of the alkaloids and control agent for strains of clinical isolates.

Strain and agent	MIC (μg/mL )			MBC (μg/mL)		
	Range	50%	90%	Range	50%	90%
*E*c-MDR ^a^						
hhS	15.63–250.0	31.25	125.0	62.50–500.0	62.50	250.0
hhC	62.50–500.0	125.0	250.0	125.00–1000.0	125.0	500.0
hS	750.0–3000.0	1500 ^d^	3000 ^d^	NA ^d^	NA ^d^	NA ^d^
hC	1500.0–3000.0	3000 ^d^	3000 ^d^	NA ^d^	NA ^d^	NA ^d^
imipenem	0.03–0.50	0.12	0.25	0.12–2.0	0.25	0.50
*K p*-MDR ^b^						
hhS	93.8–375.0	187.5	375.0	375.0–1500.0	750.0	1500.0
hhC	187.5–750.0	187.5	375.0	375.0–1500.0	750.0	1500.0
hS	750.0–3000.0	1500 ^d^	3000 ^d^	NA ^d^	NA ^d^	NA ^d^
hC	1500.0–3000.0	3000 ^d^	3000 ^d^	NA ^d^	NA ^d^	NA ^d^
imipenem	0.06–1.00	0.25	0.50	0.12–2.0	0.500	1.00
*Ec ^c^*						
hhS	15.63			31.25		
hhC	125.0			250.0		
hS	375.0			1500.0		
hC	375.0			1500.0		
imipenem	0.12			0.25		

^a^ *E*c-MDR: 18 isolates of ESBLs-producing strains from *E. coli*; ^b^ *K p*-MDR: 12 isolates of ESBLs-producing strains from *K. pneumonia*; ^c^ *Ec*: *E. coli* (ATCC25922); ^d^ NA: not active at concentrations up to 3000 μg/mL.

## 3. Experimental

### 3.1. Plant Material

Ten authenticated plant materials (Table 1) were collected in the mountainous Dali and Honghe districts of Yunnan Province, China, at attitudes of 600–2,500 m in September of 2007. They were identified by Shui Yuming of t he Kunming Institute of Botany, the Chinese Academy of Science (CAS, China) where voucher specimens were deposited.

### 3.2. Bacterial Strains and Culture Media

ESBL-producing MDR strains (18 from *E. coli* and 12 from *K. pneumoniae* isolates, respectively) were obtained from the infectious samples of critically ill patients at Kunming General Hospital (KGH). Pathogen purification and identification were conducted in the clinical microbiology laboratory of KGH following the standard procedures and the criteria of the Clinical and Laboratory Standards Institute (CLSI) [11,12,13]. Briefly, the isolates were initially screened using disks of ceftazidime (30 μg) and aztreonam (30 μg), and confirmed by using both ceftazidime and cefotaxime (30 μg), alone and in combination with clavulanic acid (10 μg). An isolate of a ≥5-mm increase in a zone diameter for either antimicrobial agent tested in combination with clavulanic acid compared with its zone when tested alone [e.g., ceftazidime zone = 16 mm; ceftazidime-clavulanic acid (30/10 μg) zone = 21 mm] was identified as an ESBL-producing isolate. The control strain ATCC 25922 (*E. coli*) was obtained from the National Institute for the Control of Pharmaceutical and Biological Products (NICPBP, China). Standard Mueller-Hinton agar and broth (MHA and MHB, Tianhe Microbial Agents Co., Hangzhou, China) were used as culture media. Imipenem (Merck & Co., Inc.) was used as the positive control agent.

### 3.3. Extract Preparation and Isolation of the Active Compounds from *C. majus* Extracts

The air-dried and ground plant material (30 g) was macerated with 80% ethanol (500 mL) for 5 days, filtered and the mare was further macerated twice with the same solvent overnight and filtered after being sonicated for 30 min. The filtrates were combined and the solvent was evaporated at 40 °C under vacuum. Ten extracts were prepared as a whole (Table 1).

A larger sample (300 g) of active extract from *C. majus* (3 kg) was suspended in 600 mL of sterilized and deionated water and the suspension was successively fractionated between water and petroleum ether and AcOEt, of which the AcOEt fraction (Fr-E) showed the most activity in a bioassay-guided fractionation procedure. The fraction (25 g) was subjected to vacuum liquid chromatography over silica gel-H (500 mesh, Qingdao Haiyang Chemical Co., Ltd. Qingdao, China) and eluted with gradients of CHCl_3_–MeOH to afford two active fractions (Fr-E1 and Fr-E2), which were further purified by reverse-phase HPLC column chromatography to yield the two alkaloids **1** (15.4 mg) and **2** (8.5 mg), respectively. Two other alkaloids, dihydrosanguinarine (hS, **3**, 200 mg) and dihydrochelerythrine (hC, **4**, 80 mg), were also obtained. All the spectral data were in agreement with the literature [14,15]. Their structures are shown in Figure 1. The MIC and MBC of the four alkaloids were determined accordingly at concentrations ranged from 0.003–3.0 mg/mL (Table 2).

### 3.4. Susceptibility Test

The ten extracts were first subjected to a susceptibility test of their IZD (mm) following the previous agar well diffusion method with a slight modification [16]. The inoculums were 1.5 × 10^8^ CFU/mL suspensions and 70 μL aliquots of the extracts (50 mg/mL in 50% aqueous DMSO) were placed in 6 mm (diam.) agar wells, incubated at 35 °C for 24 h. The MIC of the extracts (concentrations ranged from 0.5–50 mg/mL in 50% DMSO) for each organism were determined by the two-fold serial broth macro dilution method with MHB in tubes [12,16,17], with a slight modification as incubated at 35 °C for 24 h and each tube contained inoculums of 5 × 10^5^ CFU/mL. All experiments were carried out in triplicate with 50% aqueous DMSO as the solvent control. The results are shown in Table 1.

## 4. Conclusions

Ten Chinese medicinal plant extracts demonstrated varying levels of activity against clinical isolates of extended-spectrum β-lactamase (ESBL)-producing strains. Four alkaloids were identified from *C. majus* extracts, with 8-hydroxydihydrosanguinarine and 8-hydroxydihydrochelerythrine being the most active compounds. Their clinical utility against infections of ESBLs producers warrant further pharmacological investigations.

## References

[1] Gniadkowski M. (2001). Evolution and epidemiology of extended-spectrum β-lactamases (ESBLs) and ESBL-producing microorganisms. Clin. Microbiol. Infect..

[2] Pitout J.D.D., Laupland K.B. (2007). Extended-spectrum β-lactamase-producing enterobacteriaceae, an emerging public health concern. Lancet Infect. Dis..

[3] Ahmad I., Aqil F. (2007). *In vitro* efficacy of bioactive extracts of 15 medicinal plants against ESβL-producing multidrug-resistant enteric bacteria. Microbiol. Res..

[4] Lai P.K., Roy J. (2004). Antimicrobial and chemopreventive properties of herbs and spices. Curr. Med. Chem..

[5] Stavri M., Piddock L.J., Gibbons S. (2007). Bacterial efflux pump inhibitors from natural sources. J. Antimicrob. Chemother..

[6] Jiangsu New Medical College (1977). *Bai-qu-cai* *Chelidonium majus L*. Dictionary of Chinese Materia Medica.

[7] Colombo M.L., Bosisio E. (1996). Pharmacological activities of *Chelidonium majus* L. (Papaveraceae).

[8] Gibbons S. (2004). Anti-staphylococcal plant natural products. Nat. Prod. Rep..

[9] Zuo G.Y., Meng F.Y., Hao X.Y., Zhang Y.L., Wang G.C., Xu G.L. (2008). Antibacterial Alkaloids from Chelidonium majus Linn (Papaveraceae) against clinical isolates of methicillin-resistant *Staphylococcus aureus*. J. Pharm. Pharmaceut. Sci..

[10] Meng F.Y., Zuo G.Y., Hao X.Y., Wang G.C., Xiao H.T., Zhang J.Q., Xu G.L. (2009). Antifungal activity of the benzo[c]phenanthridine alkaloids from *Chelidonium majus* Linn against resistant clinical yeast isolates. J. Ethnopharmacol..

[11] Clinical and Laboratory Standards Institute (2006). Performance Standards for Antimicrobial Disk Susceptibility Tests. Approved Standard.

[12] Clinical and Laboratory Standards Institute (2007). Performance Standards for Antimicrobial Susceptibility Testing—17th Informational Supplement. Approved Standard.

[13] Farmer J.J., Murray P.R., Baron E.J., Pfaller M.A., Tenover F.C., Yolken R.H. (1999). *Enterobacteriaceae*, *Introduction, Identification*. Manual of Clinical Microbiology.

[14] Cho K.M., Yoo I.D., Kim W.G. (2006). 8-Hydroxydihydrochelerythrine, 8-hydroxydihydroSanguinarine with a potent acetylcholinesterase inhibitory activity from *Chelidonium majus* L. Biol. Pharm. Bull..

[15] Navarro V., Delgado G. (1999). Two antimicrobial alkaloids from *Bocconia arborea*. J. Ethnopharmacol..

[16] Okoli A.S., Okeke M.I., Iroegbu C.U., Ebo P.U. (2002). Antibacterial activity of *Harungana madagascariensis* leaf extracts. Phytother. Res..

[17] Clinical and Laboratory Standards Institute (2006). Methods for Dilution Antimicrobial Susceptibility Tests for Bacteria that Grow Aerobically, 7th Edition; Approved Standard.

